# Fatigue resistance of zirconia crowns coated with bioactive glass

**DOI:** 10.6026/973206300210849

**Published:** 2025-04-30

**Authors:** Aditi Kanitkar, Madhulika Srivastava, Abhijeet Alok, Siri Chandana Gonabhavi, Vijaya Lakshmi Addala, Vinay Rao

**Affiliations:** 1 Department of Prosthodontics and Crown & Bridge, Bharati Vidyapeeth Deemed to be University Dental College and Hospital, Sangli, Maharashtra, India; 2 Department of Pediatric and Preventive Dentistry, Manav Rachna Dental College, Faridabad, Haryana, India; 3 Department of Dentistry, Darbhanga Medical College and Hospital, Laheriasarai, Darbhanga, Bihar, India; 4 Department of Periodontics and Implantology, G. Pulla Reddy Dental College and Hospital, Kurnool, Andhra Pradesh, India; 5 KIMS Dental College, Amalapuram, Andhra Pradesh, India; 6 Department of Conservative Dentistry, Endodontics and Aesthetic Dentistry, AMC Dental College, Ahmedabad, Gujarat, India

**Keywords:** Zirconia crowns, bioactive glass, fatigue resistance, cyclic loading, dental ceramics, mechanical properties

## Abstract

Zirconia crowns are widely used in dentistry due to their strength and biocompatibility. However, their surface limits bonding efficiency. Therefore, it is of interest to assess the effect of bioactive glass coating on fatigue resistance of zirconia crowns. Coated crowns showed significantly higher fatigue resistance (1150 ± 45 N) compared to uncoated ones (950 ± 50 N, p < 0.05). SEM analysis confirmed reduced microcracks and better stress distribution in coated crowns. Bioactive glass coating enhances the clinical durability and performance of zirconia crowns under functional loads.

## Background:

Market entry Zirconia-based dental crowns completed widespread adoption because of their outstanding mechanical characteristics which include exceptional durability and wear-resistance alongside biocompatibility [1]. The combination of excellent occlusion resistance and aesthetic properties helps Zirconia crowns surpass metal-ceramic restorations because metal exposure and porcelain chipping along with long-term deterioration affects metal-ceramic restorations routinely [2, 3]. Fire-resistant zirconia restorations experience difficulty with tissue integration and cement and adhesive bonding because their nonbonding characteristics create a challenge [4]. Long-term failure of the restoration occurs because microleakage combined with marginal debonding and secondary caries formation at the margins [5]. Different surface modification methods are studied by researchers in order to make zirconia surface bioactive while still maintaining its structural integrity. Bioactive glass coatings provide a promising method of surface modification for zirconia because they establish strong chemical bonds with both hard and soft tissues. Silicate-based bioactive glass behaves when applied to biological fluids because it exchanges ions while establishing hydrogen bonds with natural tissues that improve restoration connections [6, 7].

Scientists have shown that bioactive glass coatings provide better zirconia-to-cementing agent adherence and establish a biological tissue-conductive atmosphere resulting in improved implant stability and both minimized bacteria adhesion and better soft tissue binding [8, 9]. The research community actively examines the effects of bioactive glass coatings on zirconia crown fatigue resistance potential despite their demonstrated benefits to zirconia bio integration. Long-term dental restoration success depends heavily on fatigue resistance because patients' crowns endure repeated masticatory cycles that trigger microcrack development and therefore result in failure [10]. The susceptibility to fatigue failure represents the main cause of prosthetic failure in medical environments particularly for patients who engage in bruxism or experience increased masticatory loads [11, 12]. A bioactive glass layer when added to zirconia crowns would produce simultaneous effects on their mechanical characteristics. The surface defects and changes in stress pattern because of the coating might decrease fatigue resistance. This protective barrier attribute of the coating spreads occlusal forces uniformly to stop both initial cracking and subsequent propagation [13, 14]. Therefore, it is of interest to evaluate the fatigue resistance of zirconia crowns coated with bioactive glass and compare them with uncoated zirconia crowns.

## Materials and Methods:

The production of 40 zirconia crowns took place with CAD/CAM technology. The study featured two crown groups totalling forty items with Group a containing twenty zirconia crowns as well as Group B that included twenty bioactive glass-coated zirconia crowns. High-translucency yttria-stabilized zirconia blocks served to produce the zirconia crowns which were sintered at 1,500°C in accordance with manufacturer standards. Bioactive glass coating application through the dip-coating methodology was used for Group B. The mixture contained four chemical compounds: SiO_2_ along with CaO and Na_2_O alongside P_2_O_5_. The bioactive glass suspension soaked zirconia crowns that received sintering at 800°C for ten minutes enabled integrated coating formation on their surface. Testing of all specimens took place using a computer-controlled universal testing machine during cyclic loading. A cyclic testing protocol with a maximum load of 200 N at 2 Hz applied 500,000 times tested the crowns that previously received resin-based luting cement and epoxy resin dies alignment. The fracture resistance evaluation relied on universal testing machine testing for measuring compressive load-to-failure on crowns following fatigue testing. The force application utilized a stainless steel indenter at the central fossa of the crowns through the universal testing machine at 1 mm/min speed until the crowns fractured. Scanning electron microscopy (SEM) was utilized to investigate the extent and ways fractures propagated while determining how the microstructure maintained its structure following the assessment of failure modes. The profilometer measured smoothness of surface textures from coated and uncoated crowns. An analysis of the data took place through SPSS software version 26. The independent t-test evaluated the fatigue resistance values between groups at p < 0.05 significance. The authors presented descriptive statistics through mean and standard deviation for each experimental group.

## Results:

The different zirconia crown coatings produced divergent results regarding their resistance to fatigue damage. The uncoated zirconia crowns (Group A) had a mean fatigue resistance of 950 ± 50 N yet the bioactive glass-coated zirconia crowns (Group B) reached a significantly increased mean fatigue resistance value of 1150 ± 45 N (p < 0.05) (Table 1). Survey results showed that 85% of zirconia crowns from Group a survived cyclic loading but this percentage increased to 95% in Group B. An evaluation of zirconia crown fracture resistance took place after performing fatigue load tests. The experimental data showed that Group A crowns had an average fracture resistance of 1900 ± 100 N but Group B crowns exhibited stronger results with 2250 ± 85 N (p < 0.05) (Table 2). Bioactive glass coatings on zirconia crowns improve their capability to withstand maximum force from biting. The surface roughness analysis indicated bioactive glass-coated zirconia crowns measured 0.60 ± 0.08 µm and this value was greater than uncoated zirconia crowns at 0.35 ± 0.05 µm with statistical significance (p < 0.05) (Table 3). The surface roughness elevation could enhance both mechanical surface interlocking and optimized stress patterns across the surface area. Scanning electron microscopy technology revealed different failure patterns in the examined groups. Bioactive glass-coated crowns displayed better structural integrity than uncoated crowns since they demonstrated a uniform stress distribution pattern together with less microcracks and inhibited crack propagation. The study shows that the bioactive glass layer functions as a protective barrier to improve the structural composition of zirconia crowns. Bioactive glass coatings increase both zirconia crown fatigue and fracture resistance and produce small surface roughness increases that potentially benefit long-term clinical outcomes.

## Discussion:

Laboratory data shows that applying bioactive glass coatings increases the resistivity of zirconia crowns against fatigue and fractures. The mechanical performance of zirconia strengthens when bioactive glass coatings are applied because they create better stress distribution and enhanced surface properties and minimize crack propagation. The data matches previous studies which show bioactive glass coatings strengthen dental materials [1, 2]. The service lifetime of dental restorations depends heavily on their fatigue resistance abilities. According to research outcomes bioactive glass-coated zirconia crowns demonstrated superior fatigue resistance than zirconia crowns without coating. The ability of bioactive glass to modify surface energy and decrease microcrack development leads to better cyclic loading resistance [3, 4]. The evidence from various research works shows that bioactive glass coatings function as material reinforcement elements by minimizing stress concentrations and reducing microstructural defects [5, 6]. Bioactive glass coating produced significant increase in fracture resistance for zirconia crowns because it acted as an effective strengthening compound. The amalgamation of bioactive glass with zirconia forms an adhesive layer that distributes stress and slows crack movement [7, 8]. Bioactive glass coatings demonstrate excellent bioactivity for serving as an energy-absorbing layer that boosts the material's resistance to mechanical defects according to research findings [9, 10]. Bioactive glass containing phosphate and calcium ions helps create hydroxyapatite through which mechanical stability and improved adhesion properties result [11]. Bioactive glass coating increases surface roughness and such topographic change could contribute to stress redistribution. The bonding strength of zirconia to luting cement will increase when the surface becomes rougher which boosts overall restoration stability [12]. The excessive amount of surface roughness should be monitored when using this approach in clinical situations because it may affect opposing tooth wear [13]. Research has established that clinical achievement requires achieving the perfect relationship between surface roughness and mechanical competence [14]. The research outcomes demonstrate bioactive glass coatings as an appealing approach to modify zirconia crowns. Bioactive glass coatings may enhance restoration durability under functional loading because they boost mechanical performance. Further research needs to examine both long-term clinical results while studying the variation of bioactive glass composition effects alongside various application methods for coating zirconia crowns.

## Conclusion:

Bioactive glass coatings significantly enhance the fatigue and fracture resistance of zirconia crowns, making them more durable under occlusal forces. The observed improvements in mechanical properties, coupled with increased surface roughness, suggest that bioactive glass may be an effective surface treatment for zirconia-based restorations. Further research is needed to optimize the application methods and assess long-term clinical performance.

## Figures and Tables

**Table 1:** Fatigue resistance values

**Group**	**Mean Fatigue Resistance (N)**	**Standard Deviation (N)**	**Survival Rate (%)**
Uncoated Zirconia (Group A)	950	50	85
Bioactive Glass-Coated Zirconia (Group B)	1150	45	95

**Table 2:** Fracture resistance values

**Group**	**Mean Fracture Resistance (N)**	**Standard Deviation (N)**
Uncoated Zirconia (Group A)	1900	100
Bioactive Glass-Coated Zirconia (Group B)	2250	85

**Table 3:** Surface roughness analysis

**Group**	**Mean Surface Roughness (µm)**	**Standard Deviation (µm)**
Uncoated Zirconia (Group A)	0.35	0.05
Bioactive Glass-Coated Zirconia (Group B)	0.6	0.08
